# Overview of renal osteodystrophy in Brazil: a cross-sectional study

**DOI:** 10.1590/2175-8239-JBN-2022-0146en

**Published:** 2023-05-08

**Authors:** Cinthia E.M. Carbonara, Noemi A.V. Roza, Luciene M. dos Reis, Aluízio B. Carvalho, Vanda Jorgetti, Rodrigo Bueno de Oliveira

**Affiliations:** 1 Universidade Estadual de Campinas, Faculdade de Ciências Médicas, Divisão de Nefrologia, Campinas, SP, Brazil.; 2 Universidade de São Paulo, Faculdade de Medicina, Departamento de Medicina Interna, Laboratório de Fisiopatologia Renal, São Paulo, SP, Brazil.; 3 Universidade Federal de São Paulo, São Paulo, SP, Brazil.; 4 Universidade Estadual de Campinas, Faculdade de Ciências Médicas da Campinas, Laboratório para o Estudo do Metabolismo Mineral e Ósseo em Nefrologia, Campinas, Campinas, SP, Brazil.

**Keywords:** Renal Insufficiency, Chronic, Chronic Kidney Disease-Mineral and Bone Disorder, Insuficiência Renal Crônica, Distúrbio Mineral e Ósseo na Doença Renal Crônica

## Abstract

**Introduction:**

The epidemiologic profile of renal osteodystrophy (ROD) is changing over time and cross-sectional studies provide essential information to improve care and health policies. The *Brazilian Registry of Bone Biopsy* (REBRABO) is a prospective, nationalmulticenter cohort that includes patients with chronic kidney disease (CKD) undergoing bone biopsy. REBRABO aims to provide clinical information on ROD. The main objective of this subanalysis was to describe the profile of ROD, including clinically relevant associations.

**Methods:**

From Aug/2015 to Dec/2021, 511 patients with CKD who performed bone biopsy were included in the REBRABO platform. Patients with no bone biopsy report (N = 40), GFR > 90 mL/min (N = 28), without asigned consent (N = 24), bone fragments inadequate for diagnosis (N = 23), bone biopsy indicated by a specialty other than nephrology (N = 6), and < 18 years old (N = 4) were excluded. Clinical-demographic data (e.g., age, sex, ethnicity, CKD etiology, dialysis vintage, comorbidities, symptoms, and complications related to ROD), laboratory (e.g., serum levels of total calcium, phosphate, parathormone, alkaline phosphatase, 25-hydroxyvitamin D, and hemoglobin), and ROD (e.g., histological diagnosis) were analyzed.

**Results:**

Data from 386 individuals were considered in this subanalysis of REBRABO. Mean age was 52 (42–60) years; 198 (51%) were male; 315 (82%) were on hemodialysis. Osteitis fibrosa (OF) [163 (42%)], adynamic bone disease (ABD) [96 (25%)] and mixed uremic osteodystrophy (MUO) [83 (21%)] were the most frequent diagnosis of ROD in our sample; 203 (54%) had the diagnosis of osteoporosis, 82 (56%) vascular calcification; 138 (36%) bone aluminum accumulation, and 137 (36%) iron intoxication; patients with high turnover were prone to present a higher frequency of symptoms.

**Conclusions:**

A high proportion of patients were diagnosed with OF and ABD, as well as osteoporosis, vascular calcification and clinical symptoms.

## Resumo

**Introdução:**

O perfil epidemiológico da osteodistrofia renal (OR) está mudando com o tempo e estudos transversais fornecem informações essenciais para melhorar cuidados e políticas de saúde. O *Registro Brasileiro de Biópsia Óssea* (REBRABO) é uma coorte nacional multicêntrica prospectiva que inclui pacientes com doença renal crônica (DRC) submetidos à biópsia óssea. O REBRABO visa fornecer informações clínicas sobre OR. O principal objetivo desta subanálise foi descrever o perfil da OR, incluindo associações clinicamente relevantes.

**Métodos:**

De Ago/2015 a Dez/2021, 511 pacientes com DRC que realizaram biópsia óssea foram incluídos na plataforma REBRABO. Excluíram-se os pacientes sem laudo de biópsia óssea (N = 40), TFG > 90 mL/min (N = 28), sem consentimento assinado (N = 24), fragmentos ósseos inadequados para diagnóstico (N = 23), biópsia óssea indicada por especialidade que não a nefrologia (N = 6), e < 18 anos de idade (N = 4). Foram analisados dados clínico-demográficos (por exemplo, idade, sexo, etnia, etiologia da DRC, tempo da diálise, comorbidades, sintomas e complicações relacionadas à OR), laboratoriais (níveis séricos de cálcio total, fosfato, paratormônio, fosfatase alcalina, 25-hidroxivitamina D e hemoglobina), e OR (diagnóstico histológico).

**Resultados:**

Dados de 386 indivíduos foram considerados nesta subanálise do REBRABO. A idade média foi 52 (42-60) anos; 198 (51%) eram homens; 315 (82%) estavam em hemodiálise. Osteíte fibrosa (OF) [163 (42%)], doença óssea adinâmica (DOA) [96 (25%)] e osteodistrofia urêmica mista (OUM) [83 (21%)] foram os diagnósticos mais frequentes de OR na amostra; 203 (54%) apresentaram diagnóstico de osteoporose, 82 (56%) calcificação vascular; 138 (36%) acúmulo ósseo de alumínio, e 137 (36%) intoxicação por ferro; pacientes com remodelação óssea aumentada eram propensos a apresentar maior frequencia de sintomas.

**Conclusões:**

Uma alta proporção de pacientes foi diagnosticada com OF e DOA, assim como osteoporose, calcificação vascular e sintomas clínicos.

## Introduction

Renal osteodystrophy (ROD) is a common complication of chronic kidney disease (CKD) associated with bone fractures, vascular calcification, and decreased quality of life^1,2,3^. In the last 40 years, the availability of new drugs and improvements in dialysis treatments have changed the epidemiologic profile of ROD^4,5,6^. Some authors observed a dominant prevalence of adynamic bone disease (ABD), while others reported a predominance of osteitis fibrosa (OF)^7,8^.

The report of case series and cohorts involving patients with ROD depict geography and ethnic differences^7,8^, which may also be related to disparities in treatment access and heterogeneous standards of quality in the provided care^9,10,11^. Regional information related to ROD may be important to support changes in health policies and to recognize important local patterns.

The *Brazilian Registry of Bone Biopsy* (REBRABO) is a prospective, national multicenter cohort that aims to provide clinical information on ROD^12^. This brief communication represents an update from previously published data from REBRABO^8^. The main objective of this subanalysis was to describe the profile of ROD, including clinically relevant associations. The secondary objective was to explore regional differences in ROD.

## Methods

This study was conducted as a subanalysis of REBRABO data. During the period from August 2015 to December 2021, 511 patients with CKD who performed a bone biopsy were included in REBRABO. Exclusion criteria were: no bone biopsy report (N = 40), GFR > 90 mL/min (N = 28), withoutsigned consent (N = 24), bone fragments inadequate for diagnosis (N = 23), bone biopsy indicated by a specialty other than nephrology (N = 6), and <18 years old (N = 4). The local ethics committee approved the study protocol (CAAE 4131141.6.0000.5404), and the research activities being reported are consistent with the Declaration of Helsinki.

All clinical, demographic and laboratory data were collected in reference to the date of bone biopsy using standard electronic forms available inthe REBRABO web system. The baseline data were entered by a nephrologist who performed the bone biopsy and validated by a single researcher. The following data were considered: age, sex, ethnicity, CKD etiology, dialysis vintage and modality, comorbidities, symptoms and complications related to ROD, drugs related to CKD-MBD, serum levels of total calcium, phosphate, parathormone, alkaline phosphatase, 25-hydroxyvitamin D, and hemoglobin. We considered the recommended range for serum levels as follows: calcium (8.8–10.2 mg/dL), phosphate (3.5–5.0 mg/dL), parathormone (≥15 – ≤65 pg/mL), and 25-vitD (30–60 ng/mL). The diagnosis of vascular calcification and bone fracture were based on information from the nephrologist who performed the bone biopsy.

Bone fragments were obtained via transiliac bone biopsies using an electrical trephine after prelabeling with tetracycline (3 days) administered over two separate periods. Undecalcified bone fragments were submitted to standard processing for histological studies^13^. Bone sections were stained with toluidine blue. Al bone content was identified by solochromeazurine staining, and iron was identified by Pearls staining. Al accumulation or iron intoxication was considered when ≥30% of the surface was covered. The samples from individual patients were classified as having OF, mixed uremic osteodystrophy (MUO), ABD, osteomalacia (OM), normal/minor alterations, osteoporosis, bone aluminum (Al) accumulation, and iron intoxication.

Continuous variables are reported as the means ± SDs or medians and interquartile intervals. Categorical data are reported as frequencies and percentages. The Mann-Whitney test and X^2^ test were applied for comparisons. Statistical analyses were performed using SPSS 22.0 (SPSS Inc., Chicago, IL). A two-sided p value <0.05 was considered statistically significant.

## Results

Data from 386 individuals were considered in our analysis. Patients were relatively young, 51% were male, 41% Caucasian, and 15% had diagnosis of diabetes. Detailed information is provided in Table 1.

**Table 1.** General clinical and biochemical data

	N = 386
Age (years)	52 (42–60)
Body mass index (kg/m^2^)	24.1 (22–27)
Gender (male; N, %)	198 (51)
Ethnicity (Caucasian; N, %)	160 (41)
Diabetes mellitus (N, %)	57 (15)
Prior cardiovascular disease (N, %)	36 (9)
CKD etiology (N, %)	
Hypertension	105 (27)
Chronic glomerulonephritis	94 (24)
Diabetes *mellitus*	46 (12)
Dialysis vintage (months)	84 (36–156)
Hemoglobin (g/dL)	11.5 (10–13)
Total calcium (mg/dL)	9.3 (8.6–9.9)
Phosphate (mg/dL)	4.9 (3.9–6.2)
Parathormone (pg/mL)	233 (63–783)
Alkaline phosphatase (IU/L)	124 (79–225)
25-vitamin D (ng/mL)	29 (21–38)

A total of 315 (82%) patients were on hemodialysis, 31 (8%) on peritoneal dialysis, and 40 (10%) on conservative management; 73 (19%) patients had undergone parathyroidectomy; 236 (61%) of patients were taking sevelamer hydrochloride, 104 (27%) calcium salts, 104 (27%) vitamin D receptor activators, 66 (17%) native vitamin D, and 98 (25%) cinacalcet hydrochloride.

### ROD Diagnosis

**Figure 1.**
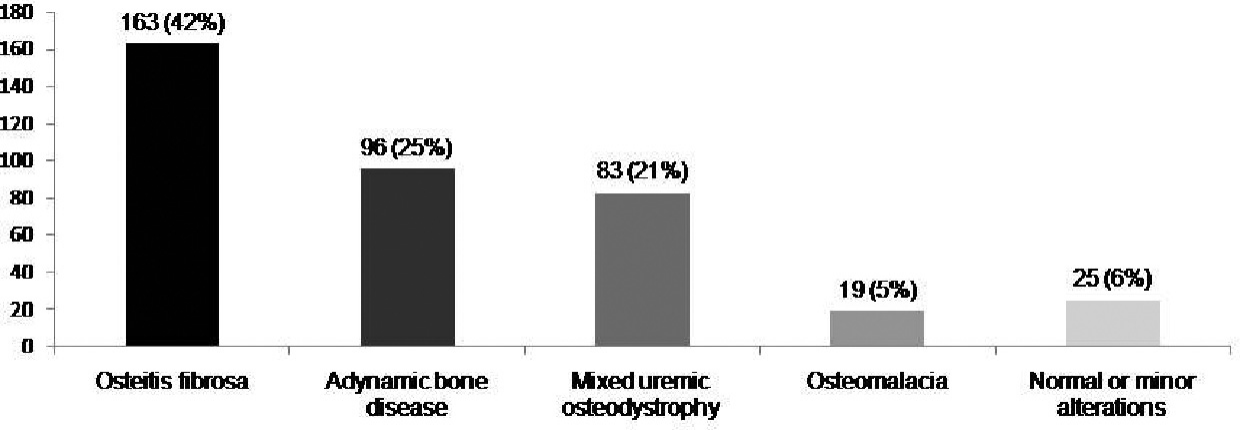
Observed frequency of renal osteodystrophy

The main indications for bone biopsy were suspicion of bone Al accumulation in 133 (34%) patients, research protocol in 114 (29%), refractory bone pain in 55 (14%), and refractory hypercalcemia/phosphatemia in 33 (8%). OF [163 (42%) patients], ABD [96 (25%) patients], and MUO [83 (21%)] were the most commonly diagnosed forms of ROD, followed by 19 (5%) patients with OM, and 25 (6%) with normal/minor alterations (Figure 1).

A total of 196 (52%) patients had abnormal bone mineralization; 203 (54%) patients had an osteoporosis diagnosis; 31 (27%) patients had low turnover bone disease and had undergone parathyroidectomy; 138 (36%) patients had a diagnosis of bone Al accumulation and 137 (36%) iron intoxication.

### Prevalence of Symptoms, Vascular Calcification and Bone Complications

A high prevalence of symptoms, vascular calcification, and bone complications was detected in our sample. Patients with high-turnover bone disease were more prone to present a higher prevalence of weakness, bone pain, myalgia, and itching than those with low turnover (Table 2). No differences in the prevalence of symptoms, vascular calcification, and bone complications were noted according to bone volume or mineralization [exception for myalgia: patients with abnormal mineralization were more prone to present high frequency of myalgia [69 (60%) *vs*. 46 (40%); p = 0.04].

**Table 2.** Prevalence of symptoms, vascular calcification, and bone complications according to bone turnover

	All	High T	Low T	p
*Clinical symptoms (N, %)*				
Weakness	163 (46)	137 (84)	26 (16)	**0.001**
Bone pain	130 (37)	103 (79)	27 (21)	**0.03**
Myalgia	108 (31)	88 (81)	20 (18)	**0.01**
Itching	54 (15)	46 (85)	8 (15)	**0.02**
*Vascular calcification (N, %)*	74 (55)	60 (81)	14 (19)	0.13
*Bone and muscular complications (N, %)*				
Bone fracture	65 (18)	47 (72)	18 (28)	0.95
Bone deformity	61 (17)	40 (66)	21 (34)	0.17
Tendon rupture	14 (4)	10 (71)	4 (29)	0.92

* T, turnover.

### Effects of ROD on Serum Biomarkers

The proportion of patients who were within the recommended range of serum levels of calcium was 54% (210 patients), of phosphate was 34% (132 patients), of parathormone was 30% (116 patients), and of 25-hydroxy vitamin D was 43% (76 patients). Hyperphosphatemia was observed in 48% (185 patients), while hypercalcemia was observed in 16% (60 patients).

Patients with high-turnover bone disease were more likely to present serum P levels outside the recommended range than patients with low turnover [186 (75%) *vs*. 61 (25%); p = 0.001]. Patients with abnormal bone mineralization were more likelyto present serum P levels outside the recommended range than those with normal mineralization [118 (47%) *vs*. 132 (53%); p = 0.007]. No other differences were noted according to bone turnover, mineralization, and volume.

### The Influence of Geographic Region on ROD

A total of 300 (78%) bone biopsies were from the Southeast region, 74 (19%) from the Northeast, 8 (2%) from the North, 3 (1%) from the Midwest, and 1 (0.3%) from the South. The type of ROD, the frequency of osteoporosis, and iron intoxication did not change according to geographic region (p = 0.08, 0.45, and 0.36, respectively).

However, we observed a distinct occurrence of bone aluminum accumulation across the regions. All bone biopsies samples from the North (8, 100%) presented aluminum accumulation, while the frequency in bone biopsies from the Northeast was 31 (42%), from the Southeast 98 (33%), and from the Midwest 1 (33%) (p = 0.02).

## Discussion

Our study shows the following findings: (1) OF and ABD were the most frequent forms of ROD; (2) osteoporosis and vascular calcification were detected in almost half of the sample, while Al and more than one-third of patients had iron deposition in bone; (3) patients with ROD, especially those with high turnover bone disease, had a high frequency of clinical symptoms; and (4) a high proportion of patients from the North and Northeast Regions had bone Al accumulation.

Compared with a previous report^8^, there was a decrease in the prevalence of OF (from 50% to 42%) and an increase in ABD (from 16% to 25%), with the prevalence of osteoporosis (from 44% to 54%) and Al accumulation (from 38% to 36%) almost maintained over time.

The high frequency of OF compared with cohorts from Europe and USA^7^ may reflect national disparities in treatment access, especially access to parathyroidectomy, and different standards of quality in provided care^14,15,16^. The high proportion of bone Al accumulation, especially in the North and Northeast, suggests the need for reinforcing strategies to avoid Al exposure, as more rigorous limits for Al concentrations in water are used for dialysis (<3 µg/L)^17^.

This study has limitations that should beacknowledge. This is an essentially descriptive study and is not a random analysis. Bone biopsy was indicated based exclusively on the referral by the Nephrologist. This study does not provide details about the indication of bone biopsy for research protocol purposes, including protocol design and inclusion or exclusion criteria. In the same way, the diagnoses of vascular calcification and bone fracture were based on information from the nephrologist who performed the bone biopsy. Laboratory data were not centered in a single unit. Sample size was limited, particularly from the North region. Our study also has strengths, as it demonstrated an elevated frequency of OF, osteoporosis, vascular calcification, clinical symptoms, and regional differences in the deposition of metals in bone in our sample.

## Conclusions

In this cohort, an elevated proportion of patients were diagnosed with OF and ABD, as well as osteoporosis, vascular calcification, and clinical symptoms. Regional differences in the deposition of metals in bone were detected and must be confirmed in future studies.
